# Effects of inbreeding on fitness-related traits in a small isolated moose population

**DOI:** 10.1002/ece3.819

**Published:** 2013-09-30

**Authors:** Hallvard Haanes, Stine S Markussen, Ivar Herfindal, Knut H Røed, Erling J Solberg, Morten Heim, Liv Midthjell, Bernt-Erik Sæther

**Affiliations:** 1 Centre for Biodiversity Dynamics, Department of Biology, Norwegian University of Science and TechnologyTrondheim, N-7491, Norway; 2 Departments of Basic Sciences and Aquatic Medicine, Norwegian School of Veterinary SciencePO-8146 Dep, Oslo, N-0033, Norway; 3 Norwegian Institute for Nature Research (NINA)Trondheim, N-7485, Norway

**Keywords:** *Alces alces*, body mass, genetic variation, inbreeding coefficients, inbreeding depression, life history traits, timing of birth, twinning rate

## Abstract

Inbreeding can affect fitness-related traits at different life history stages and may interact with environmental variation to induce even larger effects. We used genetic parentage assignment based on 22 microsatellite loci to determine a 25 year long pedigree for a newly established island population of moose with 20–40 reproducing individuals annually. We used the pedigree to calculate individual inbreeding coefficients and examined for effects of individual inbreeding (*f*) and heterozygosity on fitness-related traits. We found negative effects of *f* on birth date, calf body mass and twinning rate. The relationship between *f* and calf body mass and twinning rate were found to be separate but weaker after accounting for birth date. We found no support for an inbreeding effect on the age-specific lifetime reproductive success of females. The influence of *f* on birth date was related to climatic conditions during the spring prior to birth, indicating that calves with a low *f* were born earlier after a cold spring than calves with high *f*. In years with a warm spring, calf *f* did not affect birth date. The results suggest that severe inbreeding in moose has both indirect effects on fitness through delayed birth and lower juvenile body mass, as well as separate direct effects, as there still was a significant relationship between *f* and twinning rate after accounting for birth date and body mass as calf. Consequently, severe inbreeding as found in the study population may have consequences for population growth and extinction risk.

## Introduction

Small and isolated populations have an increased risk of extinction from genetic drift and inbreeding as opportunities for mating become restricted and the probability of mating between relatives increases (Lande 1988; Frankham 2005; Wright et al. 2008). The consequence is an increase in the frequency of homozygous genotypes (Wright 1977), which may involve inbreeding depression with reduced survival or fitness in inbred offspring (Falconer and Mackay 1996).

Inbreeding depression has been well documented through laboratory experiments and for zoo and livestock species (Wright 1977). Evidence from natural populations is increasing but involves inbreeding effects of different magnitudes as well as negative findings (Crnokrak and Roff 1999; Keller and Waller 2002; Charlesworth and Willis 2009). Inbreeding depression may for instance be reduced if detrimental and lethal recessive alleles are purged from the population (Keller and Waller 2002). Immigration may on the other hand restore genetic variation, reduce inbreeding, and increase population viability (Hedrick and Kalinowski 2000; Vilà et al. 2003), but may also re-introduce purged alleles and the positive effects may be short term (Liberg et al. 2005; Bijlsma et al. 2010; Hedrick and Fredrickson 2010). The magnitude of inbreeding depression also seems to increase with environmental stress (Keller and Waller 2002; Marr et al. 2006; Bijlsma and Loeschcke 2012). Furthermore, detection of inbreeding depression may depend on which phenotypic characters that are studied, as effects of inbreeding tend to be greater in life history traits than in morphological traits (De Rose and Roff 1999; Wright et al. 2008). Inbreeding effects on survival and reproduction have thus recently been documented in both birds (Marr et al. 2006; Grueber et al. 2010; Taylor et al. 2010; Billing et al. 2012) and mammals (Slate et al. 2000; Dunn et al. 2011; Walling et al. 2011; Olson et al. 2012). Moreover, some studies have assessed whole life spans and report separate inbreeding effects on traits at different life history stages (e.g., Szulkin et al. 2007; Grueber et al. 2010, 2011).

Many studies of inbreeding depression have been based on molecular estimates of multilocus heterozygosity in neutral markers (*MLH*), but often report weak heterozygosity-fitness correlations (HFC's) (Grueber et al. 2008; Chapman et al. 2009; Szulkin et al. 2010). However, most such HFC studies are from large and outbred populations (Grueber et al. 2008; but see e.g., Billing et al. 2012; Välimäki et al. 2007). Accordingly, the means and variances of inbreeding coefficients are usually low (Crnokrak and Roff 1999; Grueber et al. 2008, 2011), rendering a correlation between *MLH* and pedigree-calculated inbreeding (*f*) unlikely (Slate et al. 2004; Chapman et al. 2009). This emphasizes the need for studies that incorporate both molecular estimates like *MLH* and inbreeding coefficients calculated from a pedigree, preferably in populations with large mean and variance in inbreeding. Hence, more pedigree-based studies are needed to assess inbreeding in the wild and to determine how fitness-related traits are affected (Pemberton 2008; Grueber et al. 2011), especially in small and isolated populations (Grueber et al. 2008).

In this study, we show how fitness-related traits are affected by the level of individual inbreeding in the moose (*Alces alces*) population on the island Vega, off the coast of Northern Norway. Because the population was established by three founders in 1985, details on demography and life history have been established through annual radio-collaring (Sæther et al. 2003, 2007), and tissue has been sampled from almost all individuals. This provides a unique opportunity to assess the level of inbreeding in a small and isolated population and to examine its effects on fitness-related traits. Although body mass and reproduction in the population is above the Norwegian average (Solberg et al. 2011), there is substantial individual variation in fitness-related traits (Sæther et al. 2003). We hypothesize that inbreeding should have an effect on life history traits at different stages of life. Using social data and genetic parenthood, we establish a 25-year pedigree and examine the degree of inbreeding in this population, testing for effects of inbreeding on date of birth, calf body mass, twinning rate, and age-specific lifetime reproduction success. We also compare the level of pedigree-calculated inbreeding (*f*) with individual multilocus heterozygosity (*MLH*), expecting a negative relationship. Finally, we assess to what extent effects of inbreeding are modified by environmental conditions, expecting that environmental stress should increase the effects of inbreeding.

## Study Area

**Figure 1:**
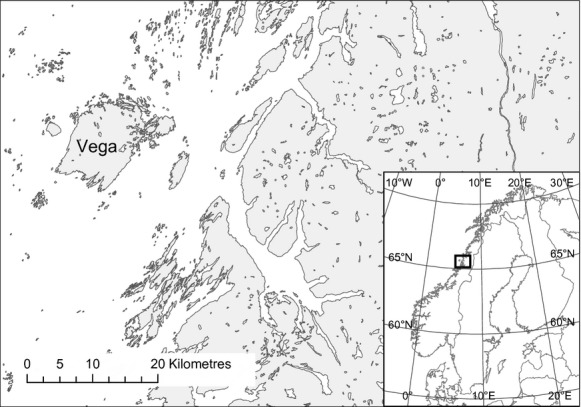
Location of the island Vega, off the coast of northern Norway.

The municipality of Vega (65º40′N, 11º55′E) consists of several islands off the coast of northern Norway (Fig 1). The main island, Vega (119 km^2^), is located approximately 13 km from the mainland. The island is relatively flat except for a mountainous part in the south-western quarter of the island that rises above the tree-line. The climate is oceanic with mild winters (mean temperature December–March: 0.5°C), cool summers (mean temperature June–August: 13°C), and a high level of precipitation (mean summer precipitation: 239 mm, winter precipitation: 394 mm with 21% as snow). The vegetation consists of coastal heath (*Calluna vulgaris*) land with open to semiopen birch (*Betula pubescens*) forest, as well as areas of Scots pine (*Pinus sylvestris*), dense Sitka spruce (*Picea sitchensis*) plantations, marshes and farmland. Most farmlands are used for grass production and for grazing cattle (Solberg et al. 2008).

**Figure 2:**
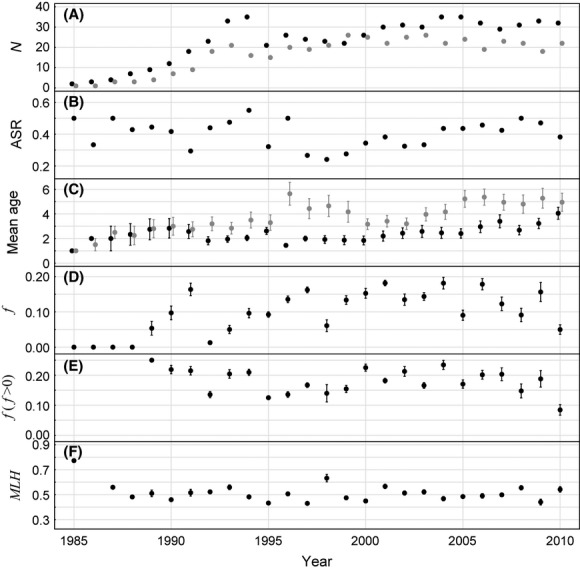
Annual variation in: (A) number of adults (age ≥ 1 years, black) and calves (gray), (B) adult sex ratio (ASR = number of bulls/number of adults), (C) mean age of adult females (gray) and males (black), (D) *f*-value of all individuals, (E) *f*-value among individuals with a positive value, (F) calf *MLH-*value. Bars show standard error of the mean.

In 1985, the island was colonized by three moose that were observed swimming to the island. By 1992, the population had increased to 24 adult individuals (Sæther et al. 2007). Harvesting started in 1989 and has from 1992 to 2010 been used to keep the number of breeding moose between 20 and 40 individuals, resulting in between 15 and 26 calves per year (Fig. 2A). The adult sex ratio has been female dominated in all years except in 1994 (Fig. 2B). Females have in general been older than males (Fig. 2C). Almost 60% of the females are usually seen in company with twins during the hunting season (Solberg et al. 2010), while triplets have not been observed. Moose hunting on the island can occur from the 25th of September to the 31st of October, but in most years started in early October, that is, after the start of the rut.

## Methods

### Sampling and social data

Sex, age, carcass mass, and tissue samples have been collected from almost all moose born on Vega that survived to the age of 4 months, that is, the start of the hunting season (Sæther et al. 2004; Solberg et al. 2010). Of 444 moose recorded during 1985–2010 (based on culling, mark and recapture, monitoring), we had tissue samples from 388 individuals (*n* = 439 samples). Three hundred and eighteen were sampled as calves, 28 as yearlings, and 42 as adults. For 20 adults with unknown age at marking or culling, the year of birth was estimated from dental cement layers (cf. Rolansen et al. 2008). All potential parents except one cow and six bulls were tissue-sampled. Each year since 1992, a social mother was determined for most calves by (1) hunter identification of the collared mother or by the location of the mother prior to and just after the calf was killed, (2) the maternal bonds observed during capturing and radiocollaring in winter (VHF/GPS), or (3) by the locations of the calf and the potential mothers in the first months after collaring, assuming that calves follow their mother. In total, we determined a social mother with fair certainty for 284 calves with tissue samples and for 46 unsampled calves (see Data S1 for further details).

### Genotyping

We used 22 microsatellite loci; CSSM03 (Moore et al. 1994), RT1, RT5, RT6, RT9, RT24, RT27, and RT30 (Wilson et al. 1997), NVHRT01, NVHRT21, and NVHRT24 (Røed and Midthjell 1998), MAF46 (Swarbrick et al. 1992), McM58, and McM64 (Hulme et al. 1994), OarFCB193 (Buchanan and Crawford 1993), BM203, BM804, BM888, BM1225, BM4107, and BM4513 (Bishop et al. 1994), and Cervid14 (DeWoody et al. 1995), of which 15 previously have been used for Norwegian moose (complete protocol cf. Haanes et al. 2011).

The genotyping error rate was calculated as the ratio between the number of differing alleles and the total number of alleles among replicated genotypes (Morin et al. 2009). For each of two groups of 40 individuals, repeated PCR's were performed and genotyped across 16 and 22 loci, respectively, and an error rate of <0.01 was estimated across loci (range = 0–0.04 per locus). Moreover, across the data set, numerous individual check-ups were carried out for separate loci after identification of apparent mismatches between (1) calves and their social mother, (2) calves and the assigned parents, (3) calves and the potential parents when none were given nor assigned, or (4) twin calves and differently assigned fathers (potential multiple paternity). Subsequent correction of discovered genotyping errors thus involved a final actual genotyping error rate that was lower than the 0.01 estimate.

### Genetic variation, parentage assignment, and pedigree

Across loci, observed and expected population heterozygosity, significance of any deviations from Hardy–Weinberg equilibrium, probabilities of null alleles and the combined probability across loci of not excluding an unrelated candidate parent with one or no parents known, were calculated with the software CERVUS 3.0 (Kalinowski et al. 2007). Allelic richness was calculated using FSTAT (Goudet 2001). Genotypic linkage disequilibrium was assessed for each yearly cohort for each pair of the 22 genotyped loci using GENEPOP with default settings (Raymond and Rousset 1995). Bonferroni correction was used to adjust for repeated tests (Rice 1989). To discover any swapped or resampled samples that might involve a double-appearance, the default identity analysis of CERVUS was run among all genotyped individuals, testing the sexes separately in a minimum of 8 loci with fuzzy matching allowing up to two mismatches.

For each parentage assignment, we used 10,000 simulations and a minimum of five sampled loci. The default 0.01 value was used for the proportion of mistyped loci, according to the estimated error rate. The number of candidate mothers and fathers varied (Table S1), and simulations were performed per year with the according number of potential parents. Known unsampled potential parents were included, and to be conservative, one additional unknown potential parent of each sex was assumed. Social maternities suggested that many mothers were closely related. Using the years 1997–2003, we calculated that roughly one quarter of the potential mothers each year were related, on average by a relatedness of 0.25. We assumed that a similar degree of relatedness existed among the males, and used this in the simulations. Because of the high degrees of relatedness and genetic similarity among the potential parents, delta-values were used in simulations (Kalinowski et al. 2007). Strict confidence level was set to 95% and the relaxed one to 80%.

Parentage assignment was initially performed by treating all potential mothers as unknown and by considering all potential parent pair combinations each year. Social mothers that were part of a parent pair that assigned with confidence (>80%) were accepted as the true mother. Cows that were part of a parent pair assigning with strict confidence (>95%) were accepted as the true mother if no social mother was given or if the social mother involved mismatches in heritage that were confirmed by repeated PCR's (exclusion). Subsequently, parentage assignment was run again with the accepted mothers excluded as potential mothers in years they appeared with twins. To include as many individuals in the pedigree as possible, we made some additional assumptions described in supplementary S2. We also added results from six calves where social maternities previously had been verified and paternities assigned through fingerprinting (Sæther et al. 2004).

Among the sampled individuals, 235 assigned with strict confidence (45 without information on social maternity), among which 170 corresponded with the social mother while 10 did not. By comparison, 119 individuals assigned with relaxed confidence, among which 95 had a social mother that corresponded in 75 of the cases. After exclusion of accepted twin mothers, ten additional relaxed assignments also corresponded with the social mother while only ten involved another mother. Combined (strict and relaxed), only 20 maternities did not match the social mother. For the fifteen assumed immigrants (including the three founders) and five other individuals sampled as adults, no parent pair or maternity assigned or matched in any year, and these were subsequently treated as immigrants. For more details on parentage assignment and the pedigree, see supplement (S2).

### Inbreeding and individual heterozygosity

From the finalized pedigree, the inbreeding coefficient per animal, the *f*-value, was calculated using Pedigraph 2.4 (Garbe and Da 2008). Individual heterozygosity (*MLH*) was estimated as the proportion of heterozygous genotypes across loci for each individual. To assess for correlation in heterozygosity across loci (identity disequilibrium), we used the REMS software to estimate the parameter *g*_*2*_, and through 1000 resampling iterations, we tested whether it was significantly different from zero (David et al. 2007; Szulkin et al. 2010).

### Effects of inbreeding on fitness-related traits

We investigated whether inbreeding (*f*) and heterozygosity (*MLH*) affected the following individual fitness-related traits: birth date (day number in year), calf body mass, and cow twinning rate and age-specific lifetime reproductive success (*asLRS*). Because most individuals are culled and many die young, age was accounted for in *asLRS*. We followed a three-step approach, using Akaike's Information Criterion adjusted for small sample size (AICc, Burnham and Anderson 2002) to rank candidate models. We first assessed whether each life history trait was affected by *f* or *MLH,* or by other individual or population parameters. Second, we added climate variables and their interactions with *f* or *MLH* to the highest ranked model from step one, and again ran AICc-based model selection. We always retained the main two effects if an interaction was included in a model. Spring and summer temperatures have previously been found to be climate variables that explain a large proportion of the variation in moose body mass (Solberg et al. 1999; Herfindal et al. 2006a). We therefore tested the effects of mean temperatures in April and May (spring temperature), and June and July (summer temperature) in the models for all life history traits. As a final step, we included as explanatory variables the preceding life history traits in each of the highest ranking models for body mass, twinning rate, and *asLRS*, that is to explore whether any inbreeding effects on these traits were affected by inbreeding effects in preceding life history traits. For subsets according to data availability, we assessed the effect of birth date on calf body mass (*N* = 281), and the effects of birth date and calf body mass on twinning rate (total sample = 49 cows, subset = 30 cows) and *asLRS* (total sample = 57 cows, subset = 36 cows). Because of potential dependencies between observations due to individuals belonging to the same cohort, the same mother, or due to repeated observations per individual for analyses on twinning rate, we ran mixed models (see below for random structure for the different traits).

Birth dates were analyzed with a Gaussian error structure with year and maternal identity as random factors. The *f* and *MLH* of calves, mothers and fathers were included to search for inbreeding effects. In addition, mother parity (primiparous or multiparous), if the calf was a twin or singleton, mother and father age, population size, and adult sex ratio were included as covariates. In step two, we also included spring temperature in the year of birth and summer temperature in the previous year as explanatory variables. Spring temperature can affect birth date by its effect on foraging conditions at the end of gestation period, whereas summer temperatures the previous year may affect birth date through a potential effect on mother body condition at the time of conception and during pregnancy.

Calf body mass was measured as carcass mass for individuals shot during the autumn hunt or live body mass in winter. Because calves grow during autumn and may loose weight in winter, we adjusted body mass relative to the date of weighing (c.f. Herfindal et al. 2006b). Because calf sex and weight category (carcass or live weight) may affect body mass, we included these two variables and their interactions in all candidate models. In the models, we further tested the effects of *f* and *MLH* of calves, mothers and fathers, mother and father age, mother parity, litter size, and the population size and adult sex ratio in the calving year. As climate variables, we used spring and summer temperatures in the year of calving.

Twinning rate was analyzed for calving cows with a logistic mixed regression with logit link function (twins = 1, singleton = 0). Year and cow identity was added as random factors. In addition, we included cow *f* and *MLH*, her age and parity, and the population size at her year of birth. We also tested the interactions of *f* or *MLH* with age and parity. As climate variables, we added spring and summer temperature from the birth year of the cow.

The shape of the relationship between cow age and *asLRS* was unknown and we therefore first ran a generalized additive mixed model (gamm, Wood 2006) between *asLRS* and age with cow birth year as a random factor to explore linearity. There was a clear nonlinear relationship (edf = 3.75, F = 56.61, *P* < 0.001), which became almost linear after ln-transforming age, although the GAMM suggested a significant weak nonlinear relationship (edf = 1.72, F = 157.6, *P* < 0.001). We therefore used a generalized linear mixed model with poisson error structure and a log link function, and cow birth year as random factor. The ln-transformed cow age was included as covariate in addition to cow *f* and *MLH*, and population size at the cows' year of birth. As for the twinning rate, we included the interactions between *f* or *MLH* and cow age, and used spring and summer temperatures at the cows' year of birth as climate variables.

## Results

### Genetic variation and parentage assignment

Significant deviations from Hardy–Weinberg equilibrium were only found in two cohorts for one locus (RT6 and BM804) among the 22 applied loci. After Bonferroni correction, significant linkage disequilibrium was only found between nine pairs of loci distributed among 6 years, as compared to the 231 pairs of loci tested each of the 25 years. The probability of null alleles was <0.05 in all loci. The combined nonexclusion probability of an unrelated candidate parent was 0.03 with no parents known and <0.001 with one parent known. Expected heterozygosity ranged from 0.23 to 0.82 (mean = 0.50, SD = 0.14) and observed heterozygosity ranged from 0.22 to 0.86 (mean = 0.50, SD = 0.14). Allelic richness ranged from 2.7 to 7.7 (mean = 3.7, SD = 1.1).

### Inbreeding and individual heterozygosity

Among the 412 individuals in the pedigree, the average level of inbreeding (*f*) across years was 0.12 (SD = 0.11, Fig. 2D). An inbreeding coefficient larger than zero was observed in 286 individuals (range = 0.02–0.47, mean = 0.17, SD = 0.09, Fig. 2E). The general trend through time was an increasing degree of inbreeding and a slight decrease in heterozygosity (Fig. 2D,E). The reduction in level of inbreeding in 1992, 1998 and 2001 and after 2007 coincides with periods when most immigration took place (four immigrants 1991–1992, eight immigrants 1998–2000, and five immigrants in 2007–2008). Shortly after each of these reductions, the level of inbreeding rapidly increased again.

Individual heterozygosity (*MLH*) ranged from 0.14 to 0.86 (mean = 0.50, SD = 0.13; Fig. 2F). From heterozygosity across loci, *g*_*2*_ was estimated to be 0.03 (SD < 0.01) and significantly different from zero (*P* < 0.001), indicating correlation of *MLH* across loci and identity disequilibrium. The individual *MLH* and *f* were correlated (*r* = −0.38, *P* < 0.001). For both *f* and *MLH,* there was a correlation between offspring and parents (*f*_Calf_ vs. *f*_Mother_: *r* = 0.40, *P* < 0.001, *f*_Calf_ vs. *f*_Father_: *r* = 0.22, *P* < 0.001, *MLH*_Calf_ vs. *MLH*_Mother_: *r* = 0.25, *P* < 0.001, *MLH*_Calf_ vs. *MLH*_Father_: *r* = 0.36, *P* < 0.001).

### Effects of inbreeding on fitness-related traits

**Figure 3:**
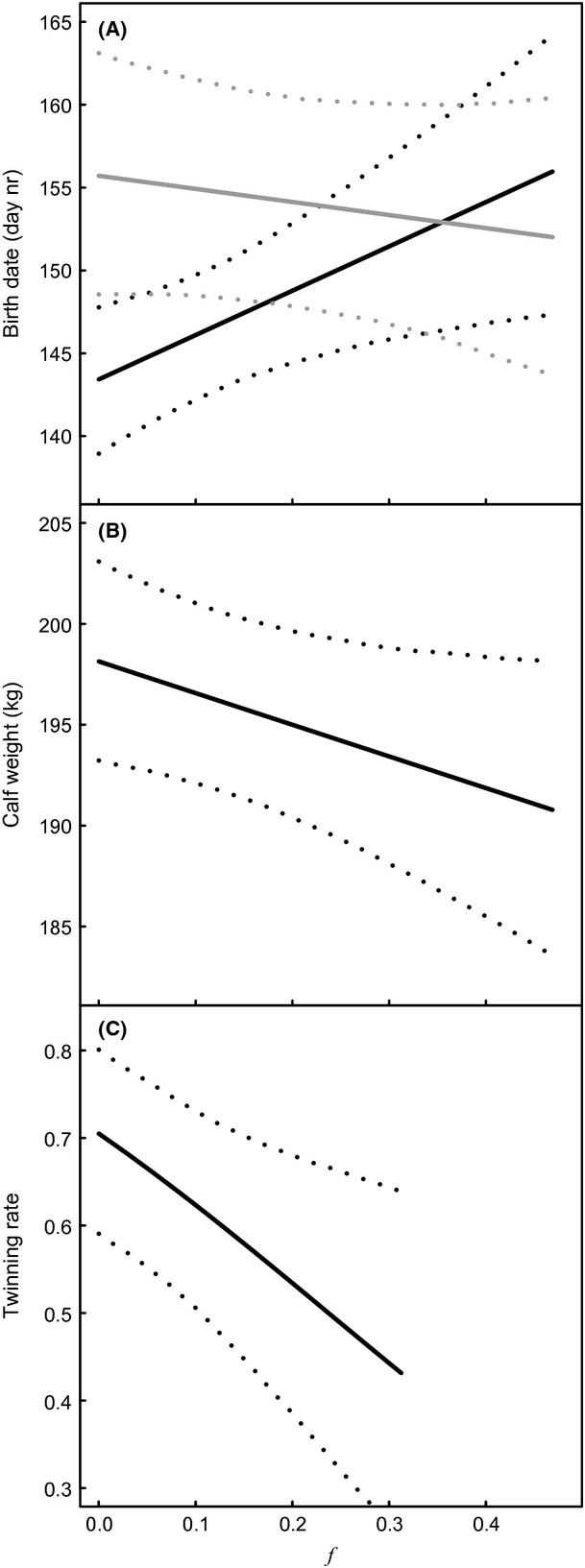
Relationship between fitness-related traits and the inbreeding coefficient (*f*) for moose on Vega. (A) Birth date and *f* for individuals born after a cold spring (mean April–May temperature = 4°C, black lines) and warm spring (mean April–May temperature = 8°C, gray lines). (B) Winter live body mass of male calves. (C) Twinning rate for calving females. Dotted lines represent 95% credible intervals based on a 10,000 MCMC resampling from the posterior distribution of the parameter estimates.

The best model explaining variation in birth date included calf *f*, population size, and whether the mother was primiparous or not (Table 1A). A model with similar support also included calf *MLH* (ΔAICc = 0.03), which parameter estimate was highly uncertain (95% CI: −2.14; 14.87). Moreover, the sum of AICc weights of candidate models including *f* was higher (0.601) compared with the sum of models including *MLH* (0.472), indicating that *f* was more important in explaining variation in birth date than *MLH*. Adding spring temperature in interaction with calf *f* increased the model fit (ΔAICc = −4.12, Table 1B). According to this model, calves with high *f* were born later than calves with low *f* following cold springs whereas no difference occurred after warm springs (interaction β = −8.07, 95% CI: −15.54; −0.32, Fig. 3A). Calves from primiparous cows were born later than from multiparous cows (estimate of difference between primiparous and multiparous: β = 9.02, 95% CI: 6.45; 12.70).

**Table 1:** AICc-based ranking of models explaining variation in individual birth date. (A) The best models considering the following individual and population parameters as explanatory variables: inbreeding coefficient (*f*) and heterozygosity (*MLH*) of the calf, its mother and its father, age of the mother and father (Age), number of siblings (1,0), mother parity (Primiparous), population size (*N*), and adult sex ratio (ASR). (B) The highest ranked models after including climate: mean temperature during April and May at birth year, *T*_Spring_, and during June and July previous year, *T*_*Summer*_. The highest ranked model (in bold) had an AICc-value of 2276.55. ΔAICc is the difference in AICc of each model relative to the highest ranked model in A. AICc weights (AICc-w) were calculated separately for model selection in A and B. For details regarding the global model and selection procedure, see Methods

Model specification	ΔAICc	AICc-w
(A)		
Primiparous + *N* + *f*	0.00	0.015
Primiparous + *N* + *f* + *MLH*	0.03	0.015
Primiparous + *N*	0.30	0.013
Primiparous + *N* + Twin + *f*	1.51	0.007
Primiparous + *N* + Age_Father_	1.51	0.007
(B)		
**Primiparous +** ***N*** **+** ***f*** ^*^ ***T***_**Spring**_	**−4.12**	**0.471**
Primiparous + *N* + *f* ^*^ *T*_Spring_ + *T*_*Summer*_	−2.01	0.163
Primiparous + *N* + *f* + *T*_Spring_	−1.74	0.143
Primiparous + *N* + *f*	0.00	0.060
Primiparous + *N* + *f* ^*^ *T*_Spring_ + *f* ^*^ *T*_*Summer*_	0.08	0.058

Calf body mass was best explained by calf *f*, mother *MLH*, mother age, mother parity (Table 2A), as well as calf sex and weight category (always retained in the models, see Methods). Again, models with *f* were given a higher support from the AICc-w (sum of AICc-w of models including *f*: 0.684) than models including *MLH* (sum of AICc-w of models including *MLH*: 0.349). Adding climate variables did not increase the fit of the highest ranked nonclimate model (ΔAICc = 0.16, Table 2B). According to the best model, calf body mass was negatively related to calf *f* (β = −15.63, 95% CI: −31.79; 1.15, Fig. 3B), and positively to mothers *MLH* (β = 13.26, 95% CI: −1.36; 28.37) and mother age (β = 1.66, 95% CI: 0.88; 2.58). Calves from primiparous cows had lower body mass than calves from multiparous cows (β = −5.22, 95% CI: −10.92; 0.59). When adding birth date to the model, the effects of calf *f* and mother *MLH* on calf body mass decreased and were associated with higher uncertainties (calf *f*: β = −12.33, 95% CI: −29.64; 3.79, mother *MLH*: β = 11.15, 95% CI: −2.67; 26.81), indicating that part of the effects of calf *f* and mother *MLH* on calf body mass is affected by birth date. Calf body mass was negatively related to birth date (β = −0.33, 95% CI: −0.49; −0.15).

**Table 2:** AICc-based ranking of candidate models explaining variation in calf body mass. (A) The best models based on individual and population parameters as explanatory variables. In addition, sex (Sex), and weight category (calf carcass mass or calf winter mass) and their interaction were always retained in the models. (B) The best models when adding climate variables to the most parsimonious model in A. The highest ranked model (in bold) had an AICc-value of 2289.19. ΔAICc is the difference in AICc of each model relative to the best model in A. AICc weights (AICc-w) were calculated separately for model selection in A and B. For details regarding the global model and selection procedure, see Methods. See Table 1 for variables explanation

Model specification	ΔAICc	AICc-w
(A)		
Weight cat^*^Sex + Primiparous + Age_**Mother**_ **+** ***f*** **+** ***MLH***_**Mother**_	**0.00**	**0.031**
Weight cat^*^Sex + Primiparous + Age_Mother_ + *f* + *f*_Mother_	0.07	0.030
Weight cat^*^Sex + Primiparous + Age_Mother_ + *f*_Mother_	0.32	0.026
Weight cat^*^Sex + Primiparous + Age_Mother_ + *f*	0.33	0.026
Weight cat^*^Sex + Primiparous + *f*_Mother_	0.78	0.021
(B)		
Weight cat^*^Sex + Primiparous + Age_Mother_ + *f* + *MLH*_Mother_	0.00	0.111
Weight cat^*^Sex + Primiparous + Age_Mother_ + *f* + *MLH*_Mother_ + *T*_*Summer*_ + *T*_Spring_	0.16	0.103
Weight cat^*^Sex + Primiparous + Age_Mother_ + *f* ^*^ *T*_*Summer*_ + *MLH*_Mother_ + *T*_Spring_	0.43	0.090
Weight cat^*^Sex + Primiparous + Age_Mother_ + *f* + *MLH*_Mother_ + *T*_Spring_	0.64	0.081
Weight cat^*^Sex + Primiparous + Age_Mother_ + *f* + *MLH*_Mother_ + *T*_*Summer*_	1.05	0.066

The twinning rate was best explained by the *f*-value of the cow, whether she was primiparous or multiparous, and population size in the year of calving. Alternative models had little support (Table 3A). Moreover, the sum of AICc weights from models including *f* (0.876) was almost twice as high compared with the sum from models including *MLH* (0.447). Adding climate variables from the cows' year of birth did not improve the fit (Table 3B). Accordingly, the twinning rate (on logit scale) was negatively related to cow *f* (β = −3.66, 95% CI: −6.52; −0.78, Fig. 3C), lower for primiparous than multiparous females (β = −1.97, 95% CI: −2.72; −1.23), and positively related to population size (β = 0.034, 95% CI: −0.001; 0.069). When adding preceding traits, there was a weak positive relationship between calf body mass and twinning rate (β = 0.023, 95%CI: −0.002; 0.049), whereas the twinning rate was negatively related to the birth date of the cow (β = −0.050, 95% CI: −0.098; −0.002). There was still a negative effect of cow *f* on twinning rate after accounting for her birth date (β = −4.90, 95% CI: −9.16; −0.69).

**Table 3:** AICc-based ranking of models explaining the variation in cow twinning rates. (A) The best models based on individual parameters and population size in the year of birth (*N*_Birth_) and year of calving (*N*). (B) The best models after including climate variables in the most parsimonious model in A. The best model had an AICc-value of 293.63. ΔAICc is the relative measure of each model relative to the best model in A. AICc weights (AICc-w) were calculated separately for model selection in A and B. See Table 1 for variables explanation

Model specification	ΔAICc	AICc-w
(A)		
**Primiparous +** ***f*** **+** ***N***	**0.00**	**0.110**
Primiparous + *f*	1.69	0.047
Primiparous^*^f + *N*	1.83	0.044
Primiparous + *f* + *N* + *N*_Birth_	1.84	0.044
Primiparous + *f* + Age + *N*	1.85	0.044
(B)		
Primiparous + *f* + *N*	0.00	0.411
Primiparous + *f* + *N* + T_Summer_	1.67	0.178
Primiparous + *f* + *N* + *T*_Spring_	2.12	0.143
Primiparous + *f* ^*^ *T*_Spring_ + *N*	3.54	0.070
Primiparous + *f* ^*^ T_Summer_ + *N*	3.76	0.067

The highest ranked model explaining the variation in *asLRS* included only cow age (Table 4A). Inclusion of cow *f* produced a model of slightly lower support (ΔAICc = 0.28), and including cow *MLH* involved even less support (ΔAICc = 1.90). The sum of AICc-w for candidate models including *f* (0.519) was again higher than for candidate models including *MLH* (0.313), but neither sum was particularly high. As we focus on inbreeding effects, we used the second best model to explore the effects of climate, but allowed model selection to exclude *f* from the candidate models. Climatic conditions in the cows' year of birth did not improve the model (ΔAICc = 0.42, Table 4B), and neither did the inclusion of birth date or calf body mass (accounting for birth date: β_*f*_ = −1.05, 95% CI −2.46; 0.32, β_Birth date_ = −0.013, 95% CI: −0.030; 0.005, accounting for calf weight: β_*f*_ = −0.95, 95% CI: −2.36; 0.43, β_Calf weight_ = 0.002, 95% CI: −0.006; 0.010). Consequently, there was no support for inbreeding effects on the *asLRS*.

**Table 4:** AICc-based ranking of candidate models explaining cow age-specific lifetime reproductive success (*asLRS)*. (A) The best models including individual parameters and population size at birth year (*N*). (B) The highest ranked models after including climate variables to the most parsimonious model in A. The best model (in bold) had an AICc-value of 62.78. ΔAICc is the difference in AICc-value a model relative to the best model in A. AICc weights (AICc-w) were calculated separately for model selection in A and B. See Table 1 for variables explanation

Model specification	ΔAICc	AICc-w
(A)		
**ln (Age)**	**0.00**	**0.241**
*f* + ln (Age)	0.29	0.209
*MLH* + ln (Age)	1.90	0.093
ln (Age) + *N*	2.32	0.076
*f* + ln (Age) + *N*	2.39	0.073
(B)		
ln (Age)	0.00	0.210
*f* + ln (Age)	0.29	0.182
ln (Age) + *T*_Spring_	0.42	0.171
*f* + ln (Age) + *T*_Spring_	1.10	0.121
ln (Age) + *T*_*Summer*_	1.81	0.085

## Discussion

### Genetic variation and inbreeding

The level of genetic variation in the small and isolated moose population on Vega was much lower in allelic richness (*A*_R_ mean = 3.7, SD = 1.1) and heterozygosity (*H*_E_ mean = 0.50, SD = 0.14) than recorded in the mainland Norwegian population (in the same 15 microsatellite loci, Haanes et al. 2011: *A*_R_ mean = 7.4, SD = 2.5, *H*_E_ mean = 0.66, SD = 0.13). This suggests strong genetic drift, as was expected from the few founders and subsequently low population size. However, albeit a low genetic variation, genetic parentage assignment was significant in most individuals. Interestingly, assignments with strict confidence corresponded very well with observed social maternities (95%). Also assignments with relaxed confidence corresponded with social maternities (83%) and after exclusion of accepted twin mothers, assignments with relaxed confidence corresponded even better with social maternities (92%). This high agreement between genetic and socially determined maternities provides credibility to previous ecological investigations at Vega (e.g., Sæther et al. 2003, 2004; Solberg et al. 2007).

In accordance with the low population size, the level of inbreeding calculated from the pedigree (*f*) was high. The level of inbreeding was reduced following immigration but shortly after increased again (Fig. 2). The variance in *f* was also high, as can be expected when inbred populations contain immigrants and their descendants (Reid et al. 2006). The relatedness structure subsequent to immigration also explains the correlation in *f* between offspring and each parental sex (Reid et al. 2006; Reid and Keller 2010). A low variation in *f* is often reported from wild populations (Grueber et al. 2008, 2011), which may explain the often reported weak or absent correlations between inbreeding and heterozygosity (Slate et al. 2004; Chapman et al. 2009). With a limited number of loci, random segregation in each locus may also have an effect (Slate et al. 2004; Hill and Weir 2011). We detected a negative correlation between *f* and *MLH,* indicating a sufficient mean and variance in *f* compared to the number of loci. The correlation in homozygosity across loci suggests a genome-wide effect (Szulkin et al. 2010), indicating that variation in *MLH* was due to inbreeding.

### Effects of inbreeding on fitness-related traits

We found negative effects of inbreeding on three fitness-related traits (Tables 3 and Fig. 3): (1) a later date of birth was associated with high *f*-values in calves, (2) calf body mass was negatively related to calf *f* and increased with mother *MLH*, and (3) twinning rates were lower for cows with higher *f*-values. Inbreeding may operate on different life history stages (Szulkin et al. 2007; Grueber et al. 2010) and the maintained relationships between inbreeding and both calf body mass and twin rate after accounting for preceding life history traits suggest that inbreeding has a separate effect on these traits. Surprisingly, we did not find any inbreeding effects on female age-specific lifetime reproductive success, but this was probably because of few individuals with data on *asLRS* and hence low statistical power. By comparison, significant effects of inbreeding have been found on the lifetime reproductive success in other ungulates (Slate et al. 2000) as well as in juvenile survival whereas none or only small effects have been found on date of birth and juvenile body mass (Overall et al. 2005; Dunn et al. 2011; Walling et al. 2011). Indeed, inbreeding depression seems to be stronger in traits that are closely related to fitness (De Rose and Roff 1999; Wright et al. 2008) and should therefore be expected in survival and reproduction parameters.

The later date of birth for inbred calves may have two explanations: (1) that conception occurs later in the rut for inbred than for more outbred calves and (2) that inbreeding involves a longer gestation period. Variation in conception date can occur as a result of varying cow condition at the onset of rut (Garel et al. 2009), or as a consequence of low availability of high-quality males (Mysterud et al. 2002). In female moose, fecundity, age at first reproduction, and twinning rate depend on body mass, which is an important life history trait in moose (Sæther and Haagenrud 1983; Solberg et al. 2008). Given the strong effect of juvenile body mass on adult body mass (Solberg et al. 2004, 2008), it is likely that the negative effect of inbreeding on calf body mass (Fig. 3B) is maintained into adulthood. Therefore, as the date of birth was unaffected by the level of inbreeding in mothers and fathers, we find it unlikely that inbreeding effects on cow conditions or mate choice caused the later birth dates of inbred calves. More likely, variation in gestation length can explain some variation in birth date, for example, if inbred fetuses have slower growth. Schwarts et al. (1988) reported that moose delayed birth date by 2 weeks following starvation while reindeer can shorten gestation by 2 weeks in response to delayed conception (Holand et al. 2006). Hence, there seems to be some flexibility in the length of the gestation period of ungulates.

One benefit of early birth is that calves have longer access to high-quality forage, which can have profound effects on body growth and fitness in large herbivores (e.g., White 1983). Moreover, as cold springs involve better forage and faster moose growth (Herfindal et al. 2006a), mothers may allocate more energy to fetuses in cold than warm springs. This could enable an earlier birth, as was found in outbred but not in inbred calves. Possibly, inbred calves are less able to take the advantage of such variation in mother's foraging conditions, making them more inclined to be born after a fixed gestation period than are outbred calves.

The lower body mass in autumn and winter of inbred calves may be explained by lower birth weight and/or lower weight gain after birth. Previous studies have found calf body mass to be related to birth date (Sæther et al. 2004), which may explain why birth date was included in the highest ranking model of inbreeding effects on calf body mass. Moreover, ungulate birth weight is also related to the time of birth (Coulson et al. 2003; Dunn et al. 2011; Walling et al. 2011). Early birth may thus affect body mass in moose by providing the calf with a longer period of access to fresh vegetation, or earlier born calves may simply have been born with higher body mass. However, as the effect of inbreeding was maintained even after accounting for date of birth, we believe that variation in calf body mass was at least partly related to different weight gain during spring and summer. This relationship was however affected by calves also being smaller when born by mothers with low heterozygosity (*MLH*). Hence, inbreeding effects on calves may also be affected by maternal effects, for example by the level of resources mothers allocate to the calf during gestation or lactation.

The lower twinning rate of inbred cows may be because inbreeding affects the body condition of cows, which is known to affect reproductive performance in moose (Sæther and Haagenrud 1983; Sæther and Andersen 1996; Solberg et al. 2008). Accordingly, the inbreeding effects on twinning rate could simply be the outcome of the inbreeding effects on birth date and calf mass, because individual variation in juvenile body mass is negatively related to birth date (Sæther et al. 2004) and is maintained into adulthood (Solberg et al. 2004, 2008). However, inbreeding can also affect fertility directly, either through sperm quality (Salisbury and Baker 1966) or female ovulation rate (Falconer and Roberts 1960; Doney and Smith 1968). At Vega, the twinning rate was weakly positively related to the calf mass of the mother and negatively related to her birth date, which supports the hypothesis that inbreeding effects on the twinning rate operate through the body conditions of the mother. However, there was still an effect of inbreeding after accounting for these relationships, indicating that inbreeding also has a separate effect on fertility. Indeed, similar independent effects of inbreeding on separate life history traits have also been reported in other studies (Szulkin et al. 2007; Grueber et al. 2010).

Because of population fragmentation and decline (Parmesan 2006; IPCC 2007), the potential effects of inbreeding on population growth and viability receive increasing concern (Keller and Waller 2002; Bijlsma and Loeschcke 2012; Pekkala et al. 2012). In ungulates, inbreeding depression has been documented in a few small or fragmented populations (e.g., red deer; Slate et al. 2000; Walling et al. 2011; Soay sheep; Coltman et al. 1999). Low genetic variation and jaw deformities were reported in a small and isolated red deer population (*n* = 50, Zachos et al. 2007), and increased genetic drift and inbreeding was found in small isolated populations of mountain goats (*Oreamnos americanus*, Ortego et al. 2011), alpine ibex (*Capra ibex*, Biebach and Keller 2010) and pronghorn (Dunn et al. 2011). Here, we report strong genetic drift and inbreeding depression in fitness-related traits within the small and isolated moose population on Vega. The inbreeding depression was expected to become more pronounced with increasing environmental stress (Keller and Waller 2002), but only variation in moose birth date was better explained when including climate variables. Early life history traits seem to be more sensitive to environmental stress than later appearing traits (e.g., Gaillard et al. 2000) and for that reason fetus growth and length of gestation may be more affected in harsh conditions. The high body growth, fecundity, and calf recruitment rates at Vega suggest that the island provides favorable living conditions for moose (Sæther et al. 2007), and this may explain why the climatic conditions are of little importance for the observed inbreeding effects. However, such effects could become more apparent at higher population densities or if the environment changes. The population has been harvested since 1989, but still the Vega population has so far been above the Norwegian average in body mass and reproduction (Solberg et al. 2011). Such high performance could also indicate that detrimental alleles not yet have accumulated or become fixated to any large extent.

## Supporting Information

**Table S1.** Yearly numbers of calves born and number of adult potential moose breeders (being potential parents the following year), and numbers of adults and calves culled each year on Vega between 1986 and 2010, with the included number of unsampled individuals in parentheses.

**Data S1.** Details on samples, assignments and pedigree assumptions.
