# Supplementary material for: Effects of inbreeding on fitness-related traits in a small isolated moose population
Source: Ecol Evol. 2013 Sep 30;3(12):4230–42. doi: 10.1002/ece3.819 (PMC3853567; doi:10.1002/ece3.819)
Supplement: Supplementary file 1 [file ece30003-4230-SD1.doc]

Table S1. Yearly numbers of calves born and number of adult potential moose breeders (being potential parents the following year), and numbers of adults and calves culled each year on Vega between 1986 and 2010, with the included number of un-sampled individuals in parentheses.

| Year | calves born | Cows present | Bulls present | Adults culled | Calves culled |
| --- | --- | --- | --- | --- | --- |
| 1985 |  | 1 | 1 | 0 | 0 |
| 1986 | 1 | 2 | 1 | 0 | 0 |
| 1987 | 3 | 2 | 2 (1) | 0 | 0 |
| 1988 | 3 | 4 | 3 (2) | 0 | 0 |
| 1989 | 4 | 5 | 4 (2) | 2 | 0 |
| 1990 | 7 | 7 | 5 (2) | 2 | 0 |
| 1991 | 9 | 12 (1) | 6 (1) | 1 | 1 |
| 1992 | 18 | 12 (1) | 11 (1) | 1 | 3 |
| 1993 | 21 | 13 | 19 (2) | 14 | 0 |
| 1994 | 16 | 13 | 22 (1) | 29 | 0 |
| 1995 | 15 | 12 | 9 | 7 | 4 |
| 1996 | 20 | 13 | 13 | 10 | 5 |
| 1997 | 19 | 17 | 7 | 6 | 9 |
| 1998 | 21 | 17 | 6 | 8 | 10 |
| 1999 | 26 | 15 | 7 | 7 | 12 |
| 2000 | 25 | 15 | 11 | 10 | 12 |
| 2001 | 22 | 17 | 13 | 11 | 8 |
| 2002 | 25 | 19 | 12 | 9 | 12 |
| 2003 | 26 | 18 | 12 | 11 | 12 |
| 2004 | 21 | 19 | 15 | 9 | 12 |
| 2005 | 23 | 18 | 16 (1) | 9 | 18 |
| 2006 | 19 | 16 | 15 (1) | 9 | 12 |
| 2007 | 23 | 16 | 14 (1) | 3 | 15 |
| 2008 | 22 | 14 | 17 | 10 | 16 |
| 2009 | 18 | 17 | 16 | 8 | 12 |
| 2010 | 22 | 20 | 12 | 5 | 17 |

## S2; Details on samples, assignments and pedigree assumptions

Since 1990, sex, age, carcass mass and tissue samples have been collected from almost all moose harvested on Vega (including individuals born 1985 – 1990). For the first colonising bull, an ear-sample was taken in 2011 from the prepared trophy dating back to 1990. Also, tissue was collected from moose immobilised and radio-collared during winter in the period 1992-2012 (Sæther *et al*. 2004; Solberg *et al*. 2010), except in 2003 and 2008 when no radio-collaring was conducted. All recorded mature cows were tissue-sampled except for one, which was present as a potential mother from 1992 (2.5 year old) to 1993. Most potential fathers were sampled except for six Vega-born bulls living in the years; 1986-1990, 1987-1990, 1991-1993, 1990-1991, 1992-1993 and 1993-1994 (potentially breeding year number two as a yearling), as well as one adult immigrant bull sampled in 2007. Bulls that were shot during the autumn were included as potential fathers for the calves born the year after. For twelve sampled individuals that were marked and collared as adults or sub-adults between 1992 and 1993 and eight individuals culled between 2009 and 2012, the year of birth was estimated with high certainty from dental cement layers (c.f Rolandsen *et al*. 2008). To ascertain these dental age estimates with one year margin each was verified through; 1) a likely assignment that year, 2) exclusion of other potential assignments the year before and after, and 3) lack of any assignments as a parent the year prior to the expected onset of reproduction given the estimated age. For seven individuals sampled as adults in 1992, and for three adults and 6 sub-adults sampled between 2002 and 2010, dental age estimation was not possible because teeth were not available (not sampled or animal still alive). These individuals were included as potential calves from two to ten years before sampling and as potential parents accordingly. The year of birth was for most of these individuals pinpointed through only one likely assignment as a calf one year, and support was gained from their reproductive inactiveness in any earlier years than assumed. Four females marked on Vega were subsequently recorded on the mainland and from then on excluded as potential mothers.

Among the 284 social maternities, 239 were first verified through significant assignment of parent-pairs (169 with strict confidence) without any information on maternity while ten were rejected on basis of exclusion and strict parent-pair assignment involving another potential mother. Among calves without a known social maternity, 55 mothers were first accepted through parent-pair assignment with strict confidence. In total we thus initially accepted the maternity of 305 individuals. Among these, the corresponding bull of each parent-pair was accepted as the father, except in six cases where the calf (with a social mother) had a twin assigning to another bull which also matched. In these cases the assigned bull was the son of the alternative bull assigning to the twin, but since the former could be excluded as father of the twin, the alternative father (matching both twins) was accepted.

After reducing the number of potential mothers (disregarding twin mothers), the remaining 83 individuals involved strict significant assignment in eight cases and relaxed assignment in 41 cases. In four cases an alternative father was accepted on basis of twin assignment. Among the strict cases three social mothers were verified while one was excluded. Among the relaxed cases six social mothers were verified, eight were excluded while ten other assignments to a non-social mother were rejected on basis of a matching and non-excludable social mother parent-pair, which was used in the pedigree. Thus, among 268 significant parent-pairs with an assumed social maternity, only 19 (7 %) did not match the social mother. We therefore also accepted the social maternity of 21 un-sampled calves with a sampled twin and accepted as father the bull assigned to the twin. For the fifteen suspected immigrants and five additional individuals sampled as adults, no parent-pairs or maternities assigned (negative LOD) or matched any year, and these were subsequently treated as immigrants.

For the last 15 non-significantly assigned samples, a social maternity was assumed for 11 but excluded in four cases (maternity) of which three matched a parent-pair with an alternative mother (which was accepted along with the bull). Seven social maternities were accepted on basis of four matching parent-pairs (three with significant maternity) and two significant and one matching maternities in two years when the prime bulls not were sampled (half-brothers involving the same *f*-value). In the same two years three cases without an assumed social mother involved significant maternity assignment and were accepted together with the un-sampled prime bull. In the last non-significant case the assumed social mother was excluded, no other mother assigned and the un-sampled cow that year was ascribed maternity while accepting the significant paternity assignement.

To further increase the resolution of the pedigree we made some additional assumptions to include more individuals. In the first few years a most likely parentage pair was assumed for four un-sampled calves, which were important as breeding adults in subsequent years as suggested by non-assigned calves those years. The first was a bull born the first year (1986), which only had one possible mother and father. The second was a bull born 1987, which had only the one and same potential father, while among the two potential mothers, each with one other known calf that year, we assume that the older 3.5 years old cow was the mother. Three years later, exclusion of the only sampled potential father suggested that one of these un-sampled bulls was the father of a twin pair of cow-calves, and since the inbreeding coefficient would be the same (the social mother was significantly assigned), an arbitrary choice was made among the two bulls as a father in the pedigree.

Furthermore, for four sampled calves that did not assign significantly but that matched in genotype only with the given social mother and one candidate father, these maternities were accepted. In 1990 and 1991, both following a year with two un-sampled adult bulls, four calves assigning significantly to a mother (three with a social maternity that corresponded) while not assigning or matching any of the sampled potential fathers, were arbitrary ascribed to the older of the two un-sampled bulls (both involved equal inbreeding). In 1994, one calf that neither matched the social mother nor assigned to any other sampled potential mother was ascribed to one un-sampled cow on basis of paternity assignment. Additionally, for 21 calves that died un-sampled before the yearly hunt, but which all had a sampled twin, their social mother and the assigned father of the twin was applied.
